# HOXA-AS2 promotes type I endometrial carcinoma via miRNA-302c-3p-mediated regulation of ZFX

**DOI:** 10.1186/s12935-020-01443-0

**Published:** 2020-07-31

**Authors:** Ning Song, Ying Zhang, Fanfei Kong, Hui Yang, Xiaoxin Ma

**Affiliations:** 1 Department of Obstetrics and Gynecology, Shengjing Hospital of China Medical University, Heping District Sanhao Street 36, Shenyang, 110004 China; 2 Experimental technology center of China Medical University, Shenyang, China

**Keywords:** Endometrial carcinoma, HOXA-AS2, MiRNA-302c-3p, ZFX, YKL-40

## Abstract

**Background:**

HOXA cluster antisense RNA2 (HOXA-AS2), a long-chain non-coding RNA, plays an important role in the behavior of various malignant tumors. The roles of HOXA-AS2 in endometrial cancer remain unclear.

**Methods:**

We test expression levels of HOXA-AS2, miRNA-302c-3p, the transcription factor zinc finger X-chromosomal protein (ZFX), and the chitinase-like protein YKL-40 in endometrial carcinoma by qRT-PCR and western blotting. Luciferase reporter and qRT-PCR assays were conducted to identify potential binding sites of HOXA-AS2 to miRNA-302c-3p. Cell cycle, migration and invasion ability of endometrial cancer cells were investigated using flow-cytometric analysis, CCK-8 and transwell assays, respectively.

**Results:**

HOXA-AS2 levels were significantly increased in endometrial cancer specimens compared to normal endometrial specimens. Upregulated HOXA-AS2 promoted invasion and proliferation of type I endometrial cancer cells. HOXA-AS2 silenced miRNA-302c-3p by binding to it. MiRNA-302c-3p negatively regulates ZFX and YKL-40. Thus HOXA-AS2 promotes the development of type I endometrial cancer via miRNA-302c-3p-mediated regulation of ZFX.

**Conclusions:**

These findings suggest that HOXA-AS2 can act as a new therapeutic target for type I endometrial cancer.

## Background

Despite advances in surgical, chemotherapeutic and bio-immunotherapeutic tumor treatments, cancer remains difficult to treat. Elucidation of the mechanisms of tumorigenesis may improve tumor prevention and treatment. Endometrial carcinoma is one of the most common gynecological malignancies. Although the average 5-year survival rate of endometrial cancer is 80%, the incidence of this cancer is increasing. Each year, 380,000 new cases of endometrial cancer occured and 89,000 cases died in the world [1]. A deeper understanding of the molecular mechanisms of endometrial carcinogenesis may provide a theoretical basis for better diagnosis and treatment of the disease.

Only 2% of genes encode proteins, the remaining genes are transcribed into non-coding RNAs (ncRNAs) [2]. NcRNAs are divided into two groups based on their length: short ncRNAs (< 200 nt) and long ncRNAs (lncRNAs, > 200 nt). LncRNAs regulate gene expression at multiple levels, including transcriptional and post-transcriptional levels [3]. They play important roles in the diagnosis, treatment, and prognosis of tumors [4, 5]. Roles of some lncRNAs in endometrial carcinoma have already been clarified. ASLNC0480 promotes the proliferation of endometrial carcinoma cells and inhibits apoptosis [6]. HOTAIR is highly expressed and associated with lymph node metastasis of endometrial carcinoma, and it is used for endometrial carcinoma classification. Downregulation of HOTAIR inhibits proliferation of endometrial carcinoma at the cellular and tissue levels [7]. Thus, substantial evidence indicates that lncRNAs are crucial regulators in endometrial carcinoma development and progression.

HOXA cluster antisense RNA2 (HOXA-AS2) is a 1048-bp lncRNA. It was found to inhibit apoptosis in early myelogenous leukemia cells treated with trans retinoic acid. HOXA-AS2 is located between HOXA3 and HOXA4 in the HOXA gene cluster and is expressed in NB4 early myelogenous leukemia cells and human peripheral blood midgranulocytes. Silencing of HOXA-AS2 reduced the cellular activity of granulocytes and increased apoptosis [8]. HOXA-AS2 expression is upregulated in gastric cancer and is related to tumor size and pathological stage and patient prognosis. HOXA-AS2 inhibits the expression of P21, PLK3, and DDZT3 by binding to EZH2, promotes the proliferation of and inhibites the apoptosis of tumor cells [9]. However, the roles of HOXA-AS2 in endometrial cancer remain unclear.

LncRNAs can serve as competing endogenous RNAs (ceRNAs) to regulate microRNAs (miRNAs). MiRNAs are non-coding small RNAs that can bind to complementary sequences in the 3′ UTRs of target mRNAs, and then regulate transcription. They are involved in various biological processes through regulatory networks, particularly occurrence, development, and metastasis of tumor and can serve as predictive markers for cancer treatment and prognosis [10]. The miR-302 cluster is a highly conserved vertebrate-specific gene cluster located on chromosome 4. Exogenous miRNA-302 suppressed human endometrial cancer and provided new clues for gene therapy of endometrial cancer [11]. MiRNA-302c, a member of the miRNA-302 family, directly acts on estrogen receptors in human breast cancer cells [12, 13]. Therefore, we inferred that miRNA-302c may play a similar role in endometrial cancer, which is also estrogen-dependent.

This study aimed to explore whether HOXA-AS2 has a role in endometrial cancer and whether miR-302c-3p is involved in its underlying mechanism of action.

## Materials and methods

### Patients and specimens

In total, 35 endometrial cancer specimens and 30 normal endometrial specimens were collected from patients visiting the Department of Obstetrics and Gynecology, Shengjing Hospital of China Medical University between 2016 and 2017. Normal endometrial specimens were obtained from patients with abnormal uterine bleeding treated with curettage. All specimens were histologically evaluated by experienced pathologists to make a pathological diagnosis. None of the patients had received chemotherapy or radiotherapy treatment prior to the study. This study was conducted in accordance with the Declaration of Helsinki and was approved by the Ethics Committee of Shengjing Hospital of China Medical University, and written informed consent was obtained from all participants.

### Cell culture

The human endometrial carcinoma cell line Ishikawa was kindly supplied by the Department of Pathophysiology, Beijing University. The human embryonic kidney (HEK) 293T cells were purchased from the Shanghai Institute of Cell Biology Academy of Sciences (Shanghai, China).

Ishikawa endometrial carcinoma cells were cultured in RPMI 1640 (Biological Industries, Kibbutz Beit Haemek, Israel) containing 10% fetal bovine serum (FBS), 50 IU/mL penicillin, and 50 mg/mL streptomycin (Invitrogen, Carlsbad, CA). HEK293T human embryonic kidney cells were maintained in DMEM/high-glucose medium (HyClone) supplemented with 10% FBS. Cells were maintained in a humidified incubator at 37 °C in the presence of 5% CO_2_.

### Transfection of cells

Lentiviral overexpression plasmids harboring pcDNA3.1-HOXA-AS2 and pcDNA3.1-ZFX were purchased from GenePharma (Shanghai, China). Ishikawa cells were transfected with 2 µg plasmid using Lipofectamine 3000 (Invitrogen, Carlsbad, CA) according to the manufacturer’s instructions, and were analyzed 48 h after transfection. A low-expression plasmid harboring sh-HOXA-AS2 and sh-ZFX was purchased from GenePharma and was transfected at a multiplicity of infection of 50. MiR-302c-3p mimic and inhibitor and their respective scrambled negative control (NC) RNAs were purchased from GenePharma. YKL-40 overexpression plasmid (pEX4-YKL-40), YKL-40 low-expression plasmid (sh-YKL-40), and their respective negative control DNAs were purchased from GenePharma. Plasmid and RNA oligo/inhibitor sequences are listed in Additional file 1: Table S1.

### Quantitative reverse-transcription (qRT-)PCR

Total RNA was extracted from tissue specimens or cultured cells using TRIzol reagent (Takara, Dalian, China) according to the manufacturer’s protocol. qRT-PCRs were conducted in duplicate using QuantiTect SYBR Green PCR Kit (Sangon Biotech, Shanghai, China). Glyceraldehyde-3-phosphate dehydrogenase (GAPDH) and RNU6 (U6) were used as internal controls because they were stably expressed across all groups. Expression levels (relative to controls) were quantified using the 2^−△△Ct^ method after normalization against the level of α-tubulin. All primers were purchased from Sangon Biotech. Primer sequences are listed in Additional file 2: Table S2.

### Protein extraction and western blotting

Proteins were extracted from endometrial specimens or cultured cells and homogenized in RIPA lysis buffer (SDS), 100 μg/ml phenylmethylsulfonyl fluoride, and 0.5% sodium deoxycholate in PBS) on ice. Supernatants were collected after centrifugation at 12,000*g* at 4 °C for 20 min. Protein concentrations were determined using a BCA protein assay kit (BioRad, China), and the whole lysates were mixed with 4 × SDS loading buffer (125 mmol/l Tris–HCl, 4% SDS, 20% glycerol, 100 mmol/l DTT, and 0.2% bromophenol blue) at a ratio of 1:3. Protein samples were heated at 100 °C for 5 min, separated on an SDS–polyacrylamide gels, and transferred to polyvinylidene fluoride membranes. The membranes were first probed with primary antibodies After incubation with horseradish peroxidase-conjugated secondary antibody, immunocomplexes were visualized using enhanced chemiluminescence. Images of the membranes were captured using Quantity One imaging software (Bio-Rad, Hercules, CA) and were used for densitometry using NIH ImageJ software. Primary antibodies used included anti-ZFX #PA5-78234(Invitrogen, Carlsbad, CA), anti-YKL-40#47066, and anti-β-actin#4970 (all from Cell Signaling, San Jose, CA). β-actin was used as a protein loading control. HRP-conjugated anti-rabbit#111-001-003 (Jackson ImmunoResearch Labs, West Grove, PA) was used as the secondary antibody. Images shown in the figures are representative of five individuals. Protein levels were normalized to the α-tubulin protein level and are expressed relative to experimental controls.

### Luciferase reporter assay

HEK293T cells were cotransfected with 5 nmol of identified miRNAs or scrambled negative controls (RiboBio, Guangzhou, China) along with 100 ng of a dual-luciferase reporter vector carrying the wild-type HOXA-AS2 fragment (pmiR-RB-Report™-HOXA-AS2; RiboBio), using Lipofectamine 3000 (Invitrogen) according to the manufacturer’s instructions. To assess interaction between miR-302c-3p and HOXA-AS2, mutant HOXA-AS2 (RiboBio) was added to the cotransfection system. At 48 h post transfection, luciferase activities were measured using a dual-luciferase reporter gene assay kit (Promega, Madison, WI) according to the manufacturer’s instructions.

### Transwell cell migration assay

Cells were seeded at 4 × 10^5^ cells/ml on top of polycarbonate Transwell filters coated with Matrigel in the upper chambers of BioCoat™ Invasion Chambers (BD Biosciences, Bedford, MA), and incubated at 37 °C for 24 h. Then, cells inside the upper chamber were removed with cotton swabs. Migratory and invasive cells on the lower membrane surface were fixed, stained with crystal violet, and counted in five random fields (40 ×) per well. Cell counts are expressed as the mean number of cells per field of view. Four independent experiments were conducted, and the data are presented as the mean ± standard deviation (SD).

### CCK-8 cell proliferation assay

Cells were grown in 96-well plates. In each well, 10 μl of CCK-8 reagent (Dojindo, Japan) was added, and cells were incubated at 37 °C with 5% CO_2_ for 24 h. The der. Each treatment group was assayed in triplicate at daily intervals after consecutive seeding for up to 3 days. Optical density at 450 nm was measured using a microplate rea

### Cell cycle and apoptosis analyses

For cell cycle analysis, following transfection, 10^6^ cells per group were harvested, washed in phosphate-buffered saline (PBS), and fixed in 70% ethanol at 4 °C overnight. Then, the cells were incubated in PBS containing Rnase A (at 1:50 of the system), and DNA was stained with propidium iodide (at 1:100 of the system). The proportions of cells in different phases of the cell cycle were assessed by flow cytometry (FACSCalibur; BD Biosciences, San Jose, CA). For apoptosis analysis, following transfection, cells were harvested and double-stained with Annexin APC/7-AAD (BioLegend, San Diego, CA) in the dark at room temperature for 10 min. Subsequently, the proportion of apoptotic cells was assessed by flow cytometry (FACSCalibur). Each group was analyzed in triplicate.

### In-situ hybridization

The localization of HOXA-AS2 in endometrial carcinoma tissues was determined using a HOXA-AS2 probe (Boster, Wuhan, China). ISH was performed using the ISH Kit according to the manufacturer’s instructions. Probes were diluted in hybridization buffer, denatured, and then hybridized at 60 °C overnight. The slides were blocked at 37 °C for 30 min and were visualized 3,3′-diaminobenzidine reaction. Images were digitally acquired on a microscope. The probe sequences of HOXA-AS2 were design as: (5′—CTCGCCGGACCCTGGCTTGGAGAAGTTCTGCGCTCCGCTG–3′).

### Statistical analysis

All statistical analyses were carried out using Prism 6.0 software (GraphPad, La Jolla, CA) and SPSS version 17.0 software (SPSS, Chicago, IL). The data were expressed as the mean ± standard deviation. Statistical significance was set at *P* < 0.05. All data were analyzed using Student’s two tailed *t* test.

## Results

### HOXA-AS2 is highly expressed in human endometrial carcinoma tissue and promotes the development of type I endometrial cancer cells in vitro

**Fig. 1:**
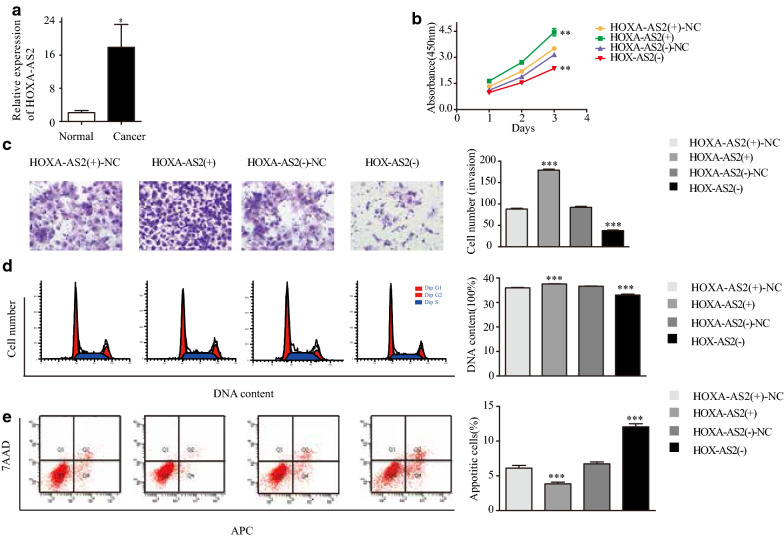
HOXA-AS2 is highly expressed in endometrial carcinoma tissues and promotes the development of type I endometrial cancer cells in vitro**. a** HOXA-AS2 mRNA levels in endometrial cancer specimens (n = 35) versus normal endometrial tissues (n = 30) as determined by qRT-PCR. **b** Cell proliferation of HOXA-AS2-modified Ishikawa cells was examined by CCK-8 assay. **c** Invasion of HOXA-AS2-modified Ishikawa cells was examined by Transwell cell invasion assays. **d** Cell cycle phases of HOXA-AS2-modified Ishikawa cells were examined by flow cytometry. **e** Fractions of apoptotic HOXA-AS2-modified Ishikawa cells were examined by flow cytometry. Data are the mean ± SD from independent experiments. *P < 0.05, ***P < 0.001

We compared HOXA-AS2 levels in 35 endometrial cancer tissues and 30 normal endometrial tissues by qRT-PCR. HOXA-AS2 levels were significantly higher in cancer tissues than in normal tissues (Fig. 1a). We discovered that HOXA-AS2 was located in the cytoplasm (Additional file 3: Fig. S1a). To investigate the function of HOXA-AS2 in endometrial cancer, we transfected Ishikawa cells with four different HOXA-AS2 siRNAs to silence HOXA-AS2. qRT-PCR for HOXA-AS2 expression analysis was conducted 48 h post transfection. Si-HOXA-AS2 2# most significantly decreased HOXA-AS2 expression (Additional file 3: Fig. S1b), therefore, was selected for subsequent experiments. In addition, overexpression of HOXA-AS2 was induced by transfecting Ishikawa cells with the pcDNA-3.1-HOXA-AS2 expression vector.

CCK-8 assays showed that Ishikawa cell growth was significantly inhibited upon HOXA-AS2 depletion, whereas it was significantly enhanced upon HOXA-AS2 overexpression (Fig. 1b). Transwell assays revealed that the invasion capability of Ishikawa cells were significantly decreased following HOXA-AS2 knockdown, but markedly increased upon HOXA-AS2 overexpression (Fig. 1c). Flow-cytometric analysis was used to determine whether the cell cycle and apoptosis of endometrial carcinoma cells were affected by HOXA-AS2. The results showed that when HOXA-AS2 knockdown, the fraction of cells in the S phase was reduced compared with that of cells transfected with NC (Fig. 1d), and the fractions of early- and late-apoptotic cells were increased compared with those of cells transfected with NC (Fig. 1d). Inversely, overexpression of HOXA-AS2 led to an increase in the S-phase cell fraction and decreases in the early- and late-apoptotic cell fractions compared to these fractions in cells transfected with the NC (Fig. 1e). These results suggested that HOXA-AS2 may act as an oncogene to promote the development and proliferation of type I endometrial carcinoma cells.

### Expression of miR-302c-3p is low in type I endometrial carcinoma cells and suppresses their development in vitro

**Fig. 2:**
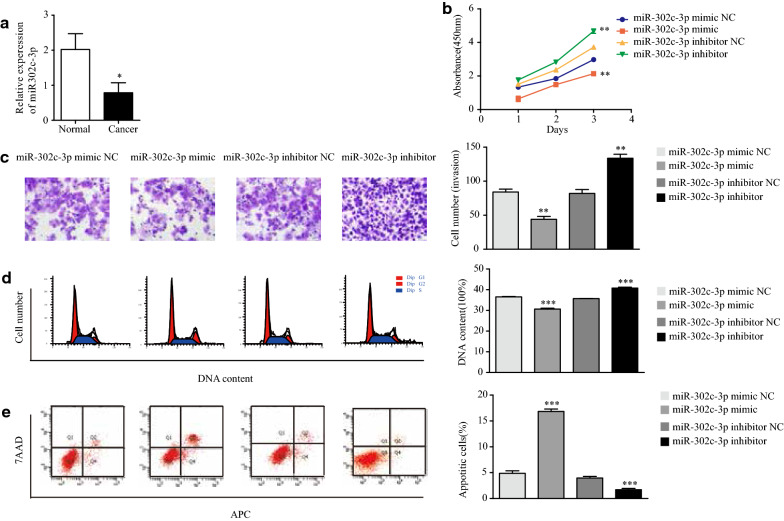
miR-302c-3p expression in endometrial carcinoma is low and suppresses the development of type I endometrial cancer cells in vitro. **a** MiR-302c-3p levels in endometrial cancer specimens (n = 35) versus normal endometrial tissues (n = 30) as determined by qRT-PCR. **b** Proliferation of miR-302c-3p-modified cells was examined by CCK-8 assay. **c** Invasion of miR-302c-3p-modified cells was examined by transwell cell invasion assays. **d** Cycle phases of miR-302c-3p-modified Ishikawa cells were examined by flow cytometry. **e** Fractions of apoptotic miR-302c-3p-modified Ishikawa cells were examined by flow cytometry. Data are the mean ± SD from independent experiments. *P < 0.05, **P < 0.01, ***P < 0.001

To investigate the role of miR-302c-3p in endometrial cancer development, miR-302c-3p expression was analyzed in 35 endometrial carcinoma samples and the 30 normal endometrial tissues by qRT-PCR. MiR-302c-3p expression was significantly downregulated in tumor tissues compared with that in normal tissues (Fig. 2a). Next, miR-302c-3p mimic and inhibitor were transfected into Ishikawa cells to induce high and low miR-302c-3p expression, respectively. CCK-8 assay revealed that the proliferation ability of endometrial cancer cells was decreased following miR-302c-3p overexpression and increased following downregulation of miR-302c-3p expression (Fig. 2b). Transwell assays indicated that the invasion capability of Ishikawa cells was significantly increased following downregulation of miR-302c-3p, but decreased following upregulation of miR-302c-3p (Fig. 2c). Flow cytometry revealed that the fraction of early- and late-apoptotic cells was decreased upon downregulation of miR-302c-3p and increased upon upregulation of miR-302c-3p (Fig. 2e). Downregulation of miR-302c-3p led to an increase in S-phase cells, whereas upregulation of miR-302c-3p led to a decrease in S-phase cells (Fig. 2d). These data suggested that miR-302c-3p may inhibit the development of endometrial cancer and acts as a suppressor of type I endometrial carcinoma cell proliferation.

### HOXA-AS2 promotes type I endometrial cancer by regulating miR-302c-3p

**Fig. 3:**
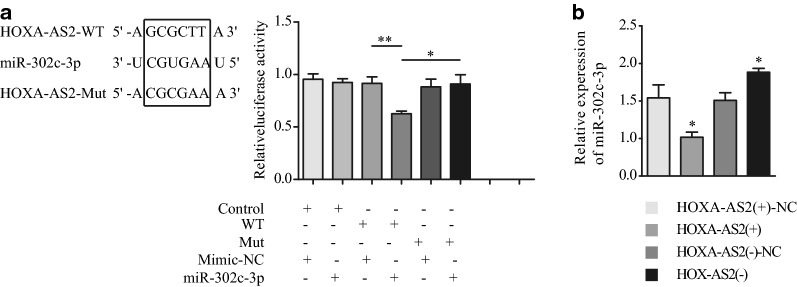
HOXA-AS2 suppresses miR-302c-3p by targeting the 3′-UTR of miR-302c -3p in Ishikawa cells. **a** Bioinformatics analyses of HOXA-AS2-target miRNAs revealed that miR-302c-3p binds to the 3′-UTR of HOXA-AS2 at the 193th–198th base site. **b** MiR-302c-3p levels in HOXA-AS2-modified Ishikawa cells as measured by qRT-PCR. *P < 0.05

Previous studies have demonstrated that HOXA-AS2 plays a catalytic role in endometrial cancer. However, the underlying mechanisms remained unclear. Using the bioinformatics database starBase v. 2.0, we determined that human HOXA-AS2 has a potential binding site for has-miR-302c-3p and, thus, may play a role in promoting endometrial cancer by regulating miR-302c-3p. To test it, we first carried out a dual luciferase assay to verify whether this binding can indeed occur and, if it does, determine the binding site. The results indicated that HOXA-AS2 could directly target miR-302c-3p via the putative binding site (Fig. 3a). Furthermore, we transfected pcDNA-3.1-HOXA-AS2 and siRNA-HOXA-AS2 into Ishikawa cells, and determined miR-302c-3p expression via qRT-PCR. MiR-302c-3p expression was significantly decreased compared to that in the control group upon HOXA-AS2 overexpression, and significantly increased compared to that in the control group upon downregulation of HOXA-AS2 (Fig. 3b). These results indicated that miR-302c-3p expression is negatively regulated by HOXA-AS2 in type I endometrial cancer.

### ZFX and YKL-40 were involved in HOXA-AS2-miR-302c-3p-induced malignant progression of type I endometrial cancer cells

**Fig. 4:**
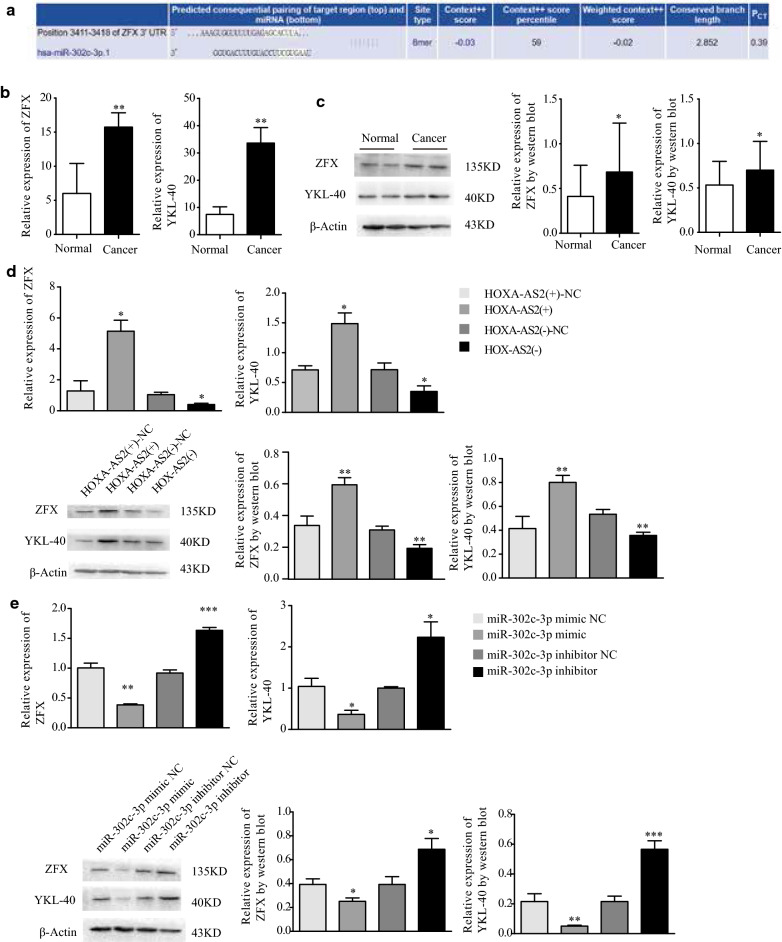
ZFX and YKL-40 are involved in the HOXA-AS2-miR-302c-3p regulatory axis. **a** the target site of ZFX and relative miR-302c-3p was confirmed by Target Scan. **b**, **c** ZFX and YKL-40 mRNA and proteins levels in endometrial cancer tissues versus normal endometrial tissues measured by qRT-PCR and western blotting. **d** ZFX and YKL-40 levels in HOXA-AS2-modified Ishikawa cells measured by qRT-PCR and western blotting. **e** ZFX and YKL-40 levels in miR-302c-3p-modified Ishikawa cells measured by qRT-PCR and western blotting. Data are the mean ± SD from independent experiments. *P < 0.05, **P < 0.01 ***P < 0.001

TargetScan predicted that miR-302c-3p has a binding site for the 3′-UTR of ZFX. (http://www.targetscan.org, Fig. 4a). The transcription factor ZFX can bind to target genes via the “GGCCT”motif. There are two “GCCT” sequences upstream of the YKL-40 transcription start site. Therefore, we infered that ZFX may be regulated by miR-302c-3p and participate in the transcriptional regulation of YKL-40 expression. Using qRT-PCR and western blotting, we found that ZFX and YKL-40 were significantly higher in endometrial cancer tissues than in normal endometrial tissues both mRNA and protein levels (Fig. 4b, c). Next, we overexpressed and downregulated HOXA-AS2 and miR-302c-3p in Ishikawa cells and evaluated ZFX and YKL-40 expression by qRT-PCR and western blotting. Compared with the control group, ZFX and YKL-40 levels were elevated when HOXA-AS2 was overexpressed and decreased when HOXA-AS2 was downregulated (Fig. 4d). Their expression was decreased when miR-302c-3p was upregulated and increased when miR-302c-3p was downregulated (Fig. 4e). These results confirmed that HOXA-AS2 and miR-302c-3p regulate ZFX and YKL-40 in type I endometrial cancer.

### ZFX promotes the development of type I endometrial cancer cells in vitro

**Fig. 5:**
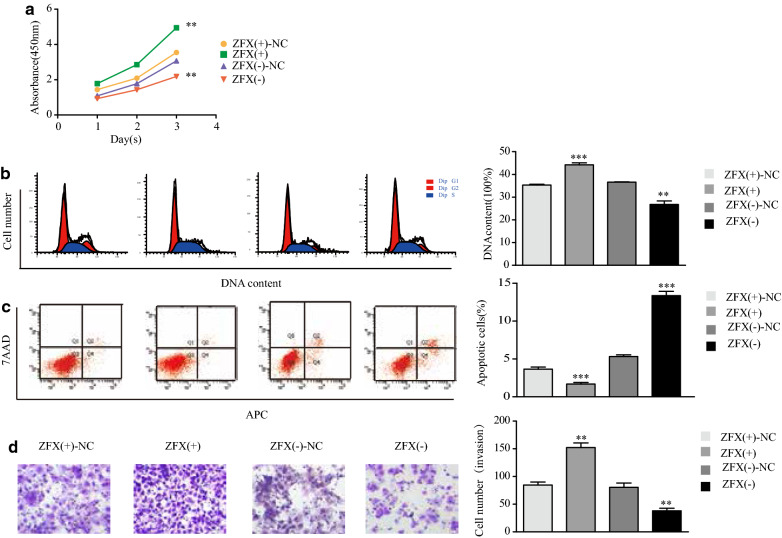
ZFX promotes the development of type I endometrial cancer cells in vitro. **a** Cell proliferation of ZFX-modified Ishikawa cells was examined by CCK-8 assay. **b** Cell cycle phases of ZFX-modified Ishikawa cells were examined by flow cytometry. **c** Fractions of apoptotic miR-302c-3p-modified Ishikawa cells were examined by flow cytometry. **d** Invasion of miR-302c-3p-modified cells was examined by Transwell invasion assays. Data are the mean ± SD from independent experiments. **P < 0.01, ***P < 0.001

It has been demonstrated that ZFX expression is higher in endometrial cancer than in normal endometrium. To elucidate the role of ZFX in endometrial cancer development further, CCK-8 assay was used to measure cell proliferation after overexpression or knockdown of ZFX in Ishikawa cells. The results indicated that cell proliferation is enhanced when ZFX is upregulated and that it is reduced when ZFX is downregulated (Fig. 5a). Flow cytometry revealed that when ZFX was upregulated, the fraction of cells in the S phase was higher than that in control group (Fig. 5b), and the fractions of cells in the early- and late-apoptotic phases were lower than those in the control group (Fig. 5c), opposite results were seen when ZFX was knocked down. Transwell assays revealed that when ZFX was upregulated, the invasive ability of endometrial cancer cells increased, whereas the invasive ability decreased following ZFX downregulation (Fig. 5d). These results indicated that ZFX promotes type I endometrial cancer development.

### YKL-40 promotes type I endometrial cell progression and is regulated by ZFX in vitro

**Fig. 6:**
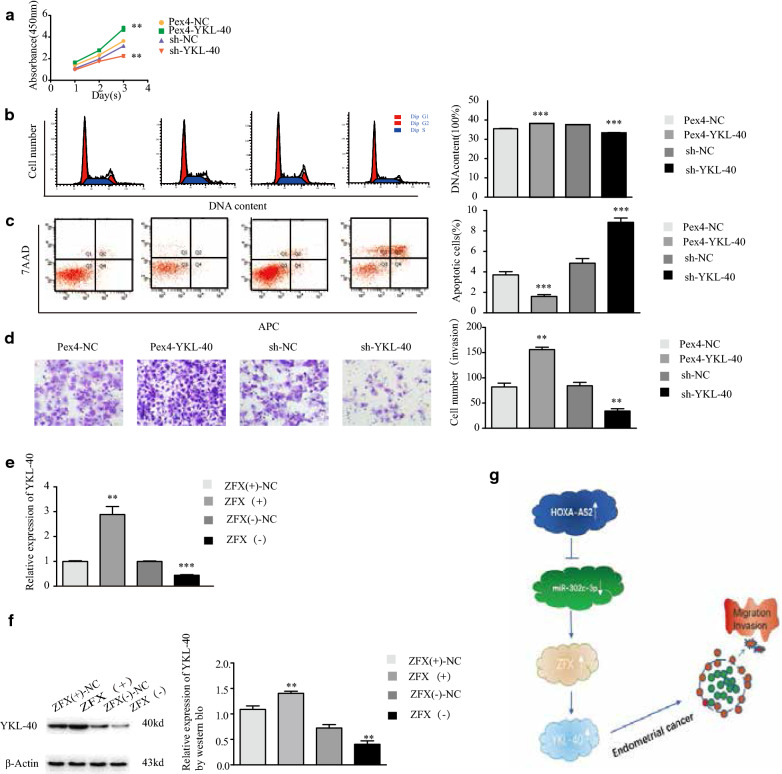
YKL-40 promotes the development of type I endometrial cancer cells and is regulated by ZFX in vitro. **a** Cell proliferation of YKL-40-modified Ishikawa cells was examined by CCK-8 assay. **b** Cell cycle phases of YKL-40-modified Ishikawa cells were examined by flow cytometry. **c** Fractions of apoptotic YKL-40-modified Ishikawa cells were examined by flow cytometry. **d** Invasion of miR-302c-3p-modified cells was examined by Transwell invasion assays. **e**, **f** YKL-40 mRNA and protein levels in ZFX-modified Ishikawa cells were measured by qRT-PCR and western blotting. Data are the mean ± SD from independent experiments. **P < 0.01, ***P < 0.001. g Proposed negative feedback loop mechanism of the HOXA-AS2-miR-302c-3p/ZFX/YKL-40 axis in Ishikawa cells. HOXA-AS2 regulate the biological behavior of the type I endometrium by negatively regulating miR-302c-3p expression. MiR-302c-3p acts as a tumor suppressor in endometrial cancer, low expression of miR-302c-3p promotes the expression of transcription factor ZFX, and high expression of ZFX promotes the expression of hydrolase YKL-40, which in turn promotes malignant behavior of type I endometrium

To validate the role of YKL-40 in endometrial cancer, YKL-40 was overexpressed and knocked down in Ishikawa cells, then,which were subjected to cell proliferation, invasion, cell cycle, and apoptosis assays. CCK-8 assay showed that Ishikawa cell proliferation was enhanced upon YKL-40 upregulation and reduced upon YKL-40 downregulation (Fig. 6a). When YKL-40 was upregulated, the fraction of cells in the S phase was higher than that in the control group (Fig. 6b), and the fractions of cells in the early- and late-apoptotic phases were lower than those in the control group (Fig. 6c). When YKL-40 was downregulated, the fraction of cells in the S phase was lower than that in the control group, and the fractions of cells in the early- and late-apoptotic phases were increased compared to those in the control group (Fig. 6b, c). Transwell assays revealed that when YKL was upregulated, the invasion ability of endometrial cancer cells increased, whereas it decreased following YKL downregulation (Fig. 6d). These results indicated that YKL promotes type I endometrial cancer development.

Next, we verified whether ZFX and YKL-40 were related. qRT-PCR and western blotting were used to detect YKL-40 expression in endometrial cancer cells with dysregulated ZFX expression. YKL-40 mRNA and protein expression were increased upon ZFX upregulation compared to that in the NC group, opposite results were seen upon downregulation of ZFX (Fig. 6e, f). These findings indicated that YKL-40 is positively regulated by ZFX in type I endometrial cancer.

## Discussion

LncRNAs, as ceRNAs, can specifically bind microRNAs,thus act as an endogenous “microRNA sponge” regulating the expression of mRNAs targeted by these microRNAs. Such a mechanism has been involved in tumor development [14]. This study demonstrated that HOXA-AS2 is highly expressed in endometrial cancer tissues and promotes the proliferation and invasion of type I endometrial cancer cells by inhibiting apoptosis. Thus, HOXA-AS2 plays an oncogenic role. On the contrary, miR-302c-3p is expressed at a low level in endometrial cancer tissues and inhibits type I endometrial cancer development. Further, our study revealed that miR-302c-3p contains a HOXA-AS2 binding site and is negatively regulated by HOXA-AS2. Thus, HOXA-AS2 may affect malignant behavior of endometrial cancer by regulating miR-302c-3p expression. Further investigation into this regulatory mechanism indicated that ZFX is highly expressed in endometrial cancer tissues and promotes the proliferation and invasion of type I endometrial cancer cells. Following upregulation of HOXA-AS2 and downregulation of miR-302c-3p, ZFX expression increased. As miR-302c-3p target, ZFX is involved in the effect of HOXA-AS2 on endometrial cancer progression. Our study also revealed that YKL-40 is positively regulated by ZFX and promotes type I endometrial cancer development.

Several studies have shown that lncRNAs play roles in malignant tumor proliferation, invasion, and metastasis and tumor resistance [15]. Some lncRNAs have been found to play a role in endometrial cancer. LncRNA FER1L4 inhibited the proliferation and invasion of endometrial cancer cells by regulating PTEN [16]. LncRNAs are currently considered an important link in the mechanism of malignant tumor development and may become important in the targeted therapy of malignant tumors [17, 18].

The 1048-bp lncRNA HOXA-AS2 was found to inhibit apoptosis in promyelocytic leukemia cells treated with retinoic acid. It is located between HOXA3 and HOXA4 in the HOXA gene cluster. HOXA-AS2 transcripts have been detected in NB4 promyelocytic leukemia cells and human peripheral blood leukocytes, and HOXA-AS2 expression is upregulated in retinoic acid-treated promyelocytic leukemia cells. Silencing of HOXA-AS2 decreased the viability and increased the apoptosis of granulocytes [8]. The role of HOXA-AS2 in malignant tumor development has attracted increasing research interest in recent years. HOXA-AS2 has been found to play a role in the development of gastric cancer, liver cancer, breast cancer, bladder cancer, intestinal cancer, and pancreatic cancer [9, 19–23] and has been reported to be related to tumor size and pathological stage and patient prognosis in gastric cancer. In gastric cancer, the expression of P21, PLK3, and DDZT3 is inhibited by binding of HOXA-AS2 to EZH2, which promotes tumor proliferation and inhibits tumor cell apoptosis [9]. Our study demonstrated that HOXA-AS2 expression is higher in endometrial cancer tissue than in normal endometrial tissue. Upregulation of HOXA-AS2 may promote cell proliferation and invasion, increase the fraction of S-phase cells, and inhibit apoptosis in type I endometrial cancer.

MiRNAs, which are mainly involved in the post-transcriptional regulation of target genes, are also involved in malignant tumor development. MiRNAs are involved in the regulation of numerous biological processes, including tumor development, and play important roles in cancer treatment and prognosis [24] Their roles differ in different tumors. Some miRNAs act as tumor suppressors, whereas others are oncogenes. For example, miR-7 inhibits the proliferation of pancreatic cancer and adrenal cortical cancer cells, acting as a tumor suppressor [25, 26]. Negrini et al. reported that miRNA-17-5p is overexpressed in gastric cancer and promotes cancer cell growth by inhibiting the activity of the oncogene-RB gene [27]. Various studies have investigated the roles of miRNAs in endometrial cancer development. Ramón et al. found that miR-210 and miR-200b are highly expressed in endometrial cancer and play a role in regulating VEGF-A [28]. Overexpression of miR-130b can promote invasion of endometrial cancer cells [29]. Increased expression of miR-200c in endometrial cancer tissues reduces the expression of BRD7, which is a potential tumor suppressor [30]. Additionally, miR-21 is overexpressed in endometrial cancer and downregulates PTEN expression, thereby promoting cell proliferation in endometrial cancer [31]. The miR-27-FOXO1 tandem induces apoptosis [32], and miR-200b inhibits TIMP2 expression and affects metastasis of endometrial adenocarcinoma [33].

The miR-302 family is a highly conserved vertebrate-specific gene cluster that was originally identified to be expressed specifically in human embryonic stem cells and human embryonic tumor cells. It play roles in stem cell pluripotency maintenance and tumor formation. In endometrial cancer cells, miRNA302 inhibits cell proliferation and migration, induces apoptosis, attenuates invasiveness by inhibiting cyclin D1 and CDK1, and arrests the cell cycle in the G2/M phase. Exogenous miRNA302 acts as a tumor suppressor in human endometrial cancer, providing new clues for gene therapy in endometrial cancer [11]. The miRNA302 family member miR-302c is highly expressed on canine and mouse embryonic stem cells and has various functions. According to recent studies, miR-302c is involved in various malignancies. It inhibits liver cancer cell proliferation and migration by targeting TRAF4 [34]. It inhibits glioma development by inhibiting cell proliferation and migration [35], and it can be used as a predictor of glioma prognosis [36]. MiR-302c/IL8 activates the RACK1-mediated receptor pathway to promote metastasis of gastric cancer [37], and low MiR-302c/IL8 expression is associated with invasion depth, tumor stage, and lymph node metastasis in gastric cancer [38]. Moreover, miR-302a/b/c/d inhibits P-glycoprotein by targeting MAP/ERK kinase 1 and thus affects doxorubicin-sensitive breast cancer metastasis [39]. Epigenetic regulation of miR302c affected the antiproliferative activity of human chondrosarcoma proline-rich polypeptides [40]. MiRNA302c can act directly on estrogen receptors in human breast cancer cells [12, 13]. In this study, miRNA-302c-3p expression was lower in endometrial cancer tissues than in normal endometrial tissues. In-vitro experiments demonstrated that high miRNA-302c-3p expression promoted apoptosis and inhibited proliferation and invasion of type I endometrial cancer cells. As such, miRNA-302c-3p may act as a tumor suppressor in type I endometrial tumors. We found that miR-302c-3p expression was negatively regulated by HOXA-AS2, indicating that HOXA-AS2 affects the biological behavior of type I endometrial cancer by interfering with miR-302c-3p expression.

ZFX, located on the X chromosome, harbors an acidic transcriptional activation domain, nuclear localization sequence, and DNA binding domains [41]. Gene transcription and translation are regulated by specific binding of regulatory molecules to target molecules, such as DNA, RNA, and DNA-RNA [42, 43]. ZFX plays a key role in controlling embryonic and hematopoietic stem cell self-renewal [44]. Considering the relationship between stem cell and tumor characteristics, the role of ZFX in tumors has received increasing research attention. Recent studies have shown that ZFX is involved in the development of many tumors [45, 46]. ZFX promotes laryngeal squamous cell carcinoma, hepatocellular carcinoma, gastric cancer, bladder cancer, and pancreatic cancer and is associated with poor patient prognosis [47–51]. ZFX can be used as a prognostic biomarker for malignant tumors [47, 50, 52]. ZFX acts as a transcriptional activator in various human malignancies mainly by binding downstream of the transcription initiation site in the CpG island promoter of target genes [53]. Recent studies have shown that miRNAs affect the biological behavior of tumors by regulating ZFX. For example, microRNA-144 affects liver cancer development by targeting ZFX [54] and microRNA-101 inhibits gallbladder carcinoma proliferation and invasion via ZFX. Our study revealed that ZFX is highly expressed in endometrial cancer and promotes endometrial cancer cell proliferation and invasion. Further, we found that miR-302c-3p exerts its effect on the biological behavior of endometrial cancer by regulating ZFX.

YKL-40, also known as human cartilage glycoprotein 39, belongs to the mammalian 18 glycosyl hydrolase family. It is mainly involved in extracellular matrix remodeling and angiogenesis, and promotes cell proliferation, migration, differentiation, and tissue remodeling processes in the inflammatory response [55]. It is also involved in the regulation of malignancy in various tumors. YKL-40 interacts with a variety of extracellular matrix components and with invasive and metastatic processes of tumor cells in lung cancer, cholangiocarcinoma, endometrial cancer, and other malignancies [56–58]. YKL-40 is an independent risk factor for the occurrence and development of various tumors [59] and has been used as a marker of tumor growth and inhibition of tumor-cell apoptosis [60]. YKL-40 has been shown to be a prognostic indicator in ovarian cancer [61–63]. Furthermore, YKL-40 seems to be involved in glandular epithelial tumors [64, 65]. In this study, YKL-40 was shown to be highly expressed in endometrial cancer tissues, promote type I endometrial cancer cell proliferation and migration, and interfere with endometrial cancer cell cycle and apoptosis. In type I endometrial cancer cells, YKL expression was regulated by ZFX, HOXA-AS2, and miR-302c-3p at both the protein and mRNA levels. YKL protein and mRNA levels were positively correlated with ZFX and HOXA-AS2 expression and negatively correlated with miR-302c-3p expression, indicating that it lies downstream of ZFX. Therefore, YKL likely participates in the development of type I endometrial cancer through the regulation of HOXA-AS2 and miR-302c-3p expression.

## Conclusions

The current study indicated that HOXA-AS2regulates the biological behavior of the type I endometrium by negatively regulating miR-302c-3p expression. MiR-302c-3p acts as a tumor suppressor in endometrial cancer and inhibits type I endometrial cancer development by regulating the transcription factor ZFX and its downstream protein YKL-40. This study revealed potential developmental mechanisms of type I endometrial cancer, which may provide new targets for the treatment of this disease.

## Supplementary information

**Additional file 1: Table S1.** Plasmid and RNA oligo/inhibitor sequences.

**Additional file 2: Table S2.** Primer sequences.

**Additional file 3: Fig.** **S1a.** HOXA-AS2 localization in endometrial carcinoma tissues.**b** si-HOXA-AS2# decreases HOXA-AS2 expression ***P < 0.001.

## Data Availability

Not applicable.

## Abbreviations

HOXA-AS2: HOXA cluster antisense RNA2
ZFX: Zinc finger X-chromosomal protein
ncRNAs: Non-coding RNAs
lncRNAs: Long ncRNAs
ceRNAs: Competing endogenous RNAs
miRNAs: MicroRNAs
