# Supplementary material for: HOXA-AS2 promotes type I endometrial carcinoma via miRNA-302c-3p-mediated regulation of ZFX
Source: Cancer Cell Int. 2020 Jul 31;20:359. doi: 10.1186/s12935-020-01443-0 (PMC7393821; doi:10.1186/s12935-020-01443-0)
Supplement: Supplementary file 1 — Additional file 1: Table S1. Plasmid and RNA oligo/inhibitor sequences. [file 12935_2020_1443_MOESM1_ESM.docx]

**Additional file 1: Table S1**

Sequences of plasmid and RNA oligo/inhibitor.

| Name | Sequence |
| --- | --- |
| Sh-HOXA-AS2-Homo-192  Sense | 5’-caccGCGCTTACCTAGAAAGATGTTTCAAGAGAACATCTTTCTAGGTAAGCGttttttg-3’ |
| Sh-HOXA-AS2-Homo-192 Antisense | 5’-gatccaaaaaaCGCTTACCTAGAAAGATGTTCTCTTGAAACATCTTTCTGGTAAGCGC-3’ |
| Sh-HOXA-AS2-Homo-433  Sense | 5’-caccGCTTTGCGTCTACAGACCTATCTTCAAGAGAGATAGGTCTGTAGACGCAAAGttttttg-3’  TTtttttg-3’ |
| Sh-HOXA-AS2-Homo-433 Antisense | 5’-gatccaaaaaaCTTTGCGTCTACAGACCTATCTCTCTTGAAGATAGGTCTGTAGACGCAAAGC-3’ |
| Sh-HOXA-AS2-Homo-623 Sense | 5’-caccGAGTTCAGCTCAAGTTGAACATTCAAGAGATGTTCAACTTGAGCTGAACTCttttttg-3’ |
| Sh-HOXA-AS2-Homo-623 Antisense | 5’-gatccaaaaaaGAGTTCAGCTCAAGTTGAACATCTCTTGAATGTTCAACTTGAGCTGAACTC-3’ |
| Sh-HOXA-AS2-Homo-734  Sense | 5’-caccGGTCAAGTATCTCAATCCAGATTCAAGAGATCTGGATTGAGATACTTGACCttttttg-3’ |
| Sh-HOXA-AS2-Homo-734 Antisense | 5’-gatccaaaaaaGCTCAAGTATCTCAATCCAGATCTCTTGAATCTGGATTGAGATACTTGACC-3’ |
| miR-Stable Negative Control | Sense: 5’-UUCUCCGAACGUGUCACGUTT-3’ |
|  | Antisense: 5’-ACGUGACACGUUCGGAGAATT-3’ |
| Has-miR-302c inhibitor | Sense: 5’-CCACUGAAACAUGGAAGCACUUA-3’ |
| Hsa-miR-302c mimic | Sense: 5’-UAAGUGCUUCCAUGUUUCAGUGG-3’ |
| miR-Inhibitor Negative Control | 5’-CAGUACUUUUGUGUAGUACAA-3’ |
| pGPU6/GFP/Neo-shNC | Sense:5’-CACCGTTCTCCGAACGTGTCACGTCAAGAGATTACGTGACACGTTCGGAGAATTTTTG-3’ |
|  | Antisense:5’-GATCCAAAAAAGTTCTCCGAACGTGTCACGTAATCTCTTGACGTGACACGTTCGGAGAAC--3’ |
| pGPU6/GFP/Neo-ZFX | Sense:5’-CACCGTCGGAAATTGATCCTTGTTTCAAGAGAACAAGGATCAATTTCCGACTTTTTTG--3’ |
|  | Antisense:5’GATCCAAAAAAGTCGGAAATTGATCCTTGTTCTCTTGAAACAAGGATCAATTTCCGAC--3’ |
| pGPU6/GFP/Neo-YKL-40 | Sense:5-CACCGACTCTCTTGTCTGTCGGATTCAAGAGATCCGACAGACAAGAGAGTCTTTTTTG--3’ |
|  | Antisense:5’GATCCAAAAAAGACTCTCTTGTCTGTCGGATCTCTTGAATCCGACAGGACAAGAGAGTC--3’ |
| pGPU6/GFP/Neo-shNC | Sense:5’-CACCGTTCTCCGAACGTGTCACGTCAAGAGATTACGTGACACGTTCGGAGAATTTTTTG-3’ |
|  | Antisense:5’-GATCCAAAAAAGTTCTCCGAACGTGTCACGTAATCTCTTGACGTGACACGTTCGGAGAAC-3’ |
