# Supplementary material for: HOXA-AS2 promotes type I endometrial carcinoma via miRNA-302c-3p-mediated regulation of ZFX
Source: Cancer Cell Int. 2020 Jul 31;20:359. doi: 10.1186/s12935-020-01443-0 (PMC7393821; doi:10.1186/s12935-020-01443-0)
Supplement: Supplementary file 2 — Additional file 2: Table S2. Primer sequences. [file 12935_2020_1443_MOESM2_ESM.docx]

**Additional file 2: Table S2**

Primer sequence.

| Gene Name | Primer Sequence |
| --- | --- |
| LINCHOXA-AS2 | F: CTGTCTGCGAAGGCCTAAAG  R: CTAGGTAAGCGCTGCTCCAA |
| hsa-miR-302c-3p | F: TAAGTGCTTCCATGTTTCAGTGG |
| ZFX | F: GCTGACCCTGGAGAAGATGA  R: TGGGAACACGAATACTGCTG |
| YKL-40 | F: GATAGCCTCCAACACCCAGA  R: TGTCTCTCCGTCCAG GGT AG |
| GAPDH | F: GCACCGTCAAGGCTGAGAAC  R: TGGTGAAGACGCCAG TTGA |
| U6 | F: GGAACGATACAGAGAAGATTAGC  R: TGGAACGCTTCACGAATTTGCG |

F: Forward; R: Reverse.
