# Supplementary figures and images for: HOXA-AS2 promotes type I endometrial carcinoma via miRNA-302c-3p-mediated regulation of ZFX
Source: Cancer Cell Int. 2020 Jul 31;20:359. doi: 10.1186/s12935-020-01443-0 (PMC7393821; doi:10.1186/s12935-020-01443-0)

a

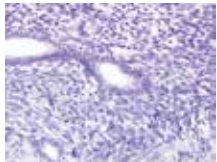

normal

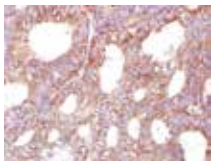

cancer

b

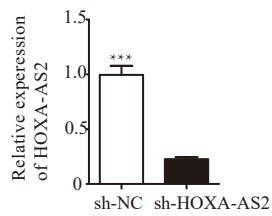

Supplement: Supplementary file 3 — Additional file 3: Fig. S1a. HOXA-AS2 localization in endometrial carcinoma tissues.b si-HOXA-AS2# decreases HOXA-AS2 expression ***P < 0.001. [file 12935_2020_1443_MOESM3_ESM.pdf]
